# Development and validation of the Executive Functioning Scale

**DOI:** 10.3389/fpsyt.2022.1078211

**Published:** 2023-01-10

**Authors:** Mirko Uljarević, Ru Ying Cai, Antonio Y. Hardan, Thomas W. Frazier

**Affiliations:** ^1^Department of Psychiatry and Behavioral Sciences, School of Medicine, Stanford University, Stanford, CA, United States; ^2^Aspect Research Centre for Autism Practice, Frenchs Forest, NSW, Australia; ^3^Department of Psychology, John Carroll University, University Heights, OH, United States

**Keywords:** emotion regulation, assessment, autism, executive functioning, working memory, response inhibition, self-regulation, neurodevelopmental

## Abstract

Executive functioning (EF) processes are essential for adaptive and flexible responding to the demands and complexities of everyday life. Conversely, if impaired, these processes are a key transdiagnostic risk factor that cuts across autism and a range of other neurodevelopmental (NDD) and neuropsychiatric (NPD) conditions. However, there are currently no freely available informant-report measures that comprehensively characterize non-affective (e.g., working memory, response inhibition, and set shifting) and affective (e.g., emotion regulation) EF subdomains. This study describes the development, refinement, and initial psychometric evaluation of a new 52-item Executive Functioning Scale (EFS). Two independent data collections yielded exploratory (*n* = 2004, 169 with autism, ages 2–17) and confirmatory (*n* = 954, 74 with autism, ages 2–17) samples. Exploratory Structural Equation Modeling (ESEM) model with six specific factors that closely matched hypothesized executive functioning subdomains of working memory and sequencing, response inhibition, set-shifting, processing speed, emotion regulation, and risk avoidance, and one general factor, showed the best fit to the data and invariance across age, sex, race, and ethnicity groups. Model reliability and internal consistency were excellent for the general factor (ω = 0.98; α = 0.97) and specific factors (ω ≥ 0.89–0.96; α ≥ 0.84–0.94). Conditional reliability estimates indicated excellent reliability (≥0.90) for the total EF scale and adequate or better reliability (≥0.70) for subscale scores. With further replication, the EFS has excellent potential for wide adoption across research and clinical contexts.

## 1. Introduction

Executive functioning (EF), emotion regulation (ER), and valuation of risk and reward are essential processes for adaptive and flexible responding to continuously shifting tasks and demands, and complexities of everyday life (1–3). Indeed, these processes underpin healthy social and emotional development (4–6) and have been associated with a range of outcomes, including academic performance (7), healthy habits (8), and different aspects of quality of life (9). Conversely, EF and ER impairments have been suggested as critical transdiagnostic risk factors that cut across a range of neurodevelopmental (NDD) and neuropsychiatric (NPD) disorders (10–14). For instance, impairments in these processes are frequent in autism spectrum disorder [ASD; (15, 16)], attention-deficit/hyperactivity disorder [ADHD; (17, 18)], schizophrenia (19, 20), depression (21, 22), obsessive-compulsive disorder [OCD; (23, 24)] and posttraumatic stress disorder (25). Further, EF and ER difficulties are associated with specific symptom domains commonly observed across NDD/NPD, including anxiety (16, 26), sameness/rituals (27, 28), social functioning difficulties (29, 30), positive and negative symptoms of psychoses (31–33) and externalizing problems (34). Thus, the existence of measures that can comprehensively evaluate noted processes that represent vital risk factors for developing and maintaining a range of clinically impactful symptoms seen across NDD and NPD is crucial for advancing etiological research and identifying treatment targets.

Although it is widely accepted that EF is best understood as a complex and multifaceted domain comprising several distinct, yet related subdomains mediated by fronto-striatal circuits (1, 35), the consensus in terms of the specific components is still lacking. More specifically, certain frameworks have focused on decontextualized and non-affective processes, emphasizing working memory, response inhibition, and set shifting, and in some instances, sequencing and planning as core EF components [e.g., (2, 36)]. Others have emphasized the need for broader conceptualization that in addition to noted “cool” EF components, also includes affective-related (or “hot”) processes, in particular, monitoring and modifying emotional responses, or ER (3, 37, 38), and risk aversion/risk-taking that encompasses evaluation of reward and punishment probability (39–41). In addition, although processing speed has not been consistently included in the definitions of EF, it has been noted that it is crucial for EF models and assessments to consider and capture processing speed given that it can underly distinct EF subdomains and has been demonstrated to show additional predictive validity regarding a range of psychopathology manifestations (42, 43). Given the noted complexity and lack of universally agreed on EF taxonomy, it is necessary for instruments to enable fine-grained capture of individual differences in a range of distinct “hot” and “cold” EF subdomains.

Several questionnaire instruments were specifically designed for assessing EF deficits in NDD and NPD. These include the Behavior Rating Inventory of Executive Functioning, second edition [BRIEF-2; (44)], the Comprehensive Executive Function Inventory [CEFI; (45)], and the Barkley Deficits in Executive Functioning Scale [BDEFS; (46, 47, 48)]. These measures have been shown to have better ecological validity compared to performance-based and experimental batteries, including, but not limited to, Delis-Kaplan Executive Function System [D-KEFS; (49)], Cambridge Neuropsychological Test Automated Battery [CANTAB; (50)], and a Developmental Neuropsychological Assessment, Second Edition [NEPSY-2; (51)], NIH Toolbox Cognition Battery (52), or the Computerized Battery for Neuropsychological Evaluation of Children [BENCI; (53)]. Further, the BRIEF-2, CEFI, and BDEFS have been extensively used across normative and a range of clinical populations, generally showing good validity and reliability. However, the above-noted instruments present a range of significant limitations. Firstly, BRIEF-2, CEFI, and BDEFS are all commercial instruments which significantly limits access and use in large-scale clinical and research collection efforts. Secondly, these measures have poor coverage and representation of specific domains. For instance, the BDEFS does not capture set-shifting. Importantly, none of the instruments capture the upregulation of positive emotions, which is a facet of ER that is just as important as the down-regulation of negative emotions (54–56) and when excessive, may also be associated with reward sensitivity and difficulties avoiding risk (57). Thirdly, there is limited evidence for the construct validity, measurement invariance, and conditional reliability of existing instruments (58). Measurement invariance is particularly important for ensuring that the measure is applicable across a broad demographic spectrum. Demonstrating good conditional reliability across a wide range of score levels is crucial for accurate assessment across neurotypical and pathological EF levels and essential for tracking change across development and due to interventions. Finally, rather than assessing everyday, developmentally appropriate behavioral instances related to specific facets of executive functioning, most available instruments assess symptoms/behavioral psychopathology thought to result from EF deficits. This focus on symptoms significantly limits the ability to capture subtle variations in functional abilities and to understand associations between these processes and specific symptom domains.

### 1.2. The present study

The present paper describes the development and preliminary psychometric evaluation of the Executive Functioning Scale (EFS)—a brief, freely available, informant-report measure specifically designed to address limitations of existing instruments and comprehensively characterize individual variation in specific, well-defined facets of EF across the normative-pathological continuum. The EFS was developed based on the recommendations for item generation and refinement outlined by the National Institute of Health’s Scientific Standards of the Patient-Reported Outcomes Measurement Information System (PROMIS) and in conjunction with NDD and NPD individuals and their parents. Detailed psychometric evaluation was conducted in two independent, large, representative samples spanning normative and atypical development and included evaluation of factor structure, measurement invariance, classical test theory and item response theory-derived reliability, and testing of convergent and discriminant validity.

## 2. Materials and methods

### 2.1. Participants

Parent informants were recruited using the Prolific online data collection service,^1^ and interested participants were directed *via* a link to the Qualtrics survey. Two separate data collections were conducted to establish exploratory and confirmatory samples. For the exploratory sample, data were collected from 03/04/2022 to 04/17/2022. A total of 2,486 informants consented and responded to the survey, with the final sample comprising 2,004 valid responses (124 respondents were excluded due to not completing the survey, 72 were excluded due to completing too rapidly to produce valid results, and 286 were excluded due to failing at least one of the four attention checks from the modified Conscientious Responders Scale [CRS; (59)]. According to informant reports, 169 children have received a diagnosis of autism spectrum disorder, 541 had other NDD/NPD, and 1,294 were neurotypical. For the confirmatory sample, data were collected from 05/03/2022 to 07/20/2022. A total of 1,361 informants consented and responded to the survey; however, given the considerable length of the survey, EFS data was collected only from 954 responders to reduce the participant burden (407 participants who did not complete the EFS completed additional instruments). Thus, the final confirmatory sample comprised 954 responses. Based on informant reports, 74 children have received a diagnosis of autism spectrum disorder, 249 had other NDD/NPD, and 631 were neurotypical. Inclusion criteria for both exploratory and confirmatory samples included: residence in the US, having a dependent child aged 2–17, and informant proficiency in English. Detailed characteristics across exploratory and confirmatory samples are presented in Table 1.

**TABLE 1:** Demographic and clinical characteristics across autism spectrum disorder (ASD), developmental disability (DD), and neurotypical (NT) controls across exploratory and confirmatory samples.

	NT	DD	ASD	*X*^2^/*F* (*p*)
	*n* (%)	*n* (%)	*n* (%)	
N	1925	790	243	
Informant (n,%)				74.86 (<0.001)
Biological mother	1119 (58.1%)	513 (64.9%)	167 (68.7%)	
Biological father	686 (35.6%)	184 (23.3%)	53 (21.8%)	
Other/Not reported	120 (6.3%)	93 (11.8%)	23 (9.5%)	
Highest parental education (n,%)				26.8 (0.003)
Less than HS	6 (0.6%)	2 (0.6%)	1 (1.0%)	
High school or GED	90 (8.9%)	38 (10.8%)	11 (10.6%)	
Some college	178 (17.6%)	94 (26.8%)	32 (30.8%)	
College graduate	427 (42.2%)	132 (37.6%)	40 (38.5%)	
Graduate degree or higher	295 (29.2%)	80 (22.8%)	18 (17.3%)	
Unknown	16 (1.6%)	5 (1.4%)	2 (1.9%)	
US region				10.9 (0.205)
Northeast	188 (18.6%)	51 (14.5%)	16 (15.4%)	
Midwest	215 (21.3%)	69 (19.7%)	23 (22.1%)	
South	402 (39.8%)	168 (47.9%)	50 (48.1%)	
West	203 (20.1%)	62 (17.7%)	15 (14.4%)	
Other/Chose not to respond	4 (0.4%)	1 (0.3%)	0 (0.0%)	
Household income (n,%)				59.2 (<0.001)
<$25,000	281 (9.5%)	92 (11.6%)	38 (15.6%)	
$25,000–$34,999	288 (9.7%)	87 (11.0%)	29 (11.9%)	
$35,000–$49,999	348 (11.8%)	96 (12.2%)	43 (17.7%)	
$50,000–$74,999	641 (21.7%)	176 (22.3%)	50 (20.6%)	
$75,000–$99,999	496 (16.8%)	140 (17.7%)	34 (14.0%)	
$100,000–$149,999	563 (19.0%)	129 (16.3%)	26 (10.7%)	
$150,000–$199,999	176 (5.9%)	36 (4.6%)	13 (5.3%)	
$200,000 and above	138 (4.7%)	28 (3.5%)	8 (3.3%)	
Unknown	27 (0.9%)	6 (0.8%)	2 (0.8%)	
Child age (M, SD)	8.58 (4.70)	11.46 (4.50)	10.31 (4.74)	111.8 (<0.001)
Child biological sex (n,% male)	915 (47.7%)	431 (54.6%)	181 (74.8%)	69.9 (<0.001)
**Race**				
White/Caucasian (n,%)	1578 (82.0%)	658 (83.3%)	200 (82.3%)	0.67 (0.716)
Black/African American (n,%)	182 (9.5%)	71 (9.0%)	29 (11.9%)	1.9 (0.385)
Middle Eastern (n,%)	5 (0.3%)	4 (0.5%)	2 (0.8%)	2.4 (0.305)
East Asian (n,%)	66 (3.4%)	11 (1.4%)	4 (1.6%)	9.9 (0.007)
South Asian (n,%)	33 (1.1%)	2 (0.1%)	2 (0.1%)	10.1 (0.006)
Pacific Islander (n,%)	10 (0.5%)	4 (0.5%)	1 (0.1%)	0.5 (0.975)
Native American (n,%)	22 (1.1%)	20 (2.5%)	6 (2.5%)	7.9 (0.019)
Multiracial (n,%)	151 (5.1%)	74 (2.5%)	27 (0.0%)	3.9 (0.139)
Unknown race (n,%)	3 (0.2%)	2 (0.3%)	0 (0.0%)	0.7 (0.683)
Chose not to respond (n,%)	15 (0.8%)	3 (0.4%)	1 (0.4%)	1.6 (0.445)
Hispanic or Latino (n,%)	101 (11.4%)	43 (12.1%)	26 (22.4%)	12.4 (0.015)
**Non-ASD diagnoses (n,%)**				
ID/GDD	–	10 (2.8%)	6 (5.8%)	2.1 (0.150)
Speech/Language disorder	–	75 (21.4%)	16 (15.5%)	1.7 (0.193)
ADHD	–	146 (41.6%)	29 (27.9%)	6.1 (0.014)
ODD/CD	–	25 (7.1%)	5 (4.9%)	0.7 (0.415)
Anxiety disorder	–	111 (31.6%)	19 (18.4%)	6.8 (0.009)
Specific learning disorder	–	33 (9.4%)	3 (2.9%)	4.6 (0.032)
Motor/Coordination disorder	–	16 (4.6%)	2 (1.9%)	1.4 (0.231)
Depressive disorder	–	50 (14.2%)	8 (1.8%)	3.0 (0.083)
Bipolar disorder/Mania	–	7 (2.0%)	1 (1.0%)	0.5 (0.488)
Obsessive compulsive disorder	–	11 (3.1%)	5 (4.9%)	0.7 (0.405)
Tic disorder	–	6 (1.7%)	1 (1.0%)	0.3 (0.593)
Feeding/Eating disorder	–	16 (4.6%)	0 (0.0%)	4.9 (0.029)

NT, neurotypical controls; DD, non-ASD developmental disability; ASD, autism spectrum disorder; ID/GDD, intellectual disability/global developmental delay; ADHD, attention-deficit/hyperactivity disorder; ODD/CD, oppositional defiant disorder/conduct disorder. Non-ASD diagnoses do not sum to 100% because children could be diagnosed with more than one condition. Cognitive level information was completed for *n* = 886.

### 2.2. Measures

#### 2.2.1. Exploratory sample

##### 2.2.1.1. Demographic and diagnostic information

Informants completed a background survey indicating informant and child age, informant and child gender, child race/ethnicity, informant relationship status, household income, and estimates of autism spectrum disorder (ASD) symptom severity and cognitive level. Informants also indicated whether the child had a clinical diagnosis of neurodevelopmental or neuropsychiatric disorder, including intellectual disability/global developmental delay, speech/language disorder, attention-deficit/hyperactivity disorder, oppositional defiant disorder/conduct disorder, anxiety disorder, specific learning disorder, motor/coordination disorder, depressive disorder, bipolar disorder/mania, OCD, tic disorder, and feeding/eating disorder. All children without ASD but with other diagnoses were recoded to a developmental disability (DD) category. Participants with no developmental or neuropsychiatric diagnosis were assigned to the neurotypical (NT) group.

##### 2.2.1.2. Executive Functioning Scale (EFS)

EFS items were developed and refined through the iterative steps described by the PROMIS framework described below.

###### 2.2.1.2.1. Conceptual model generation

Systematic literature search was performed to identify existing EF instruments and conceptual models. As noted, there is a lack of universally agreed-on EF taxonomy. Given different definitions and that a wide range of potential EF subdomains put forward across different conceptualizations is strongly related to clinical symptoms observed across NDD and NPD, we took the position that it is important to capture a broad set of distinct “hot” and “cold” EF subdomains. Identified subdomains included: working memory and sequencing, response inhibition, set-shifting, processing speed, emotion regulation, and risk avoidance.

###### 2.2.1.2.2. Item writing

Systematic review of the literature was conducted to identify existing scales relevant to each domain and content area. Scales reviewed included: (i) dedicated questionnaire measures of executive functioning such as the BRIEF, ECI, and BDEFS, (ii) dedicated experimental measures of distinct executive functioning domains such as the Dimensional Card Sorting Task, Stroop Task, as well as comprehensive testing batteries including D-KEFS (49), CANTAB (50), and a NEPSY-2 (51); (iii) general psychopathology and development instruments including the Behavior Assessment System for Children [BASC; (60)] and Infant–Toddler Social and Emotional Assessment [ITSEA; (61)], (iv) temperament and personality measures [e.g., the Infant Behavior Questionnaire (62)], and (v) measures of emotion regulation and self-regulation such as the Emotion Regulation Checklist (63) and Difficulties in Emotion Regulation Scale (64). The first and senior authors reviewed instruments, and items across identified measures were organized into specific latent constructs, then pruned and adjusted for consistency. At least three items were written to ensure that the content area is adequately assessed and that future analyses on these items could identify any sub-factors within each domain. As much as possible, items were developed not to probe more than one construct, or the endorsement of an item is not a consequence of distinct processes.

###### 2.2.1.2.3. Preliminary item evaluation and refinement

Fifty-two items were developed by the research team to evaluate each of the above executive functioning subdomains. The preliminary item bank was evaluated by ten neurodevelopmental disability clinician-scientist experts and ten neurodevelopmental disability caregiver/patient informants with regards to whether each item: (i) effectively evaluated the specific executive functioning subdomain (experts and informants), (ii) was relevant to patients (experts) or child (informants), (iii) was relevant to the full age and functional range of patients (experts), and (iv) was easy/difficult to understand (experts and informants). Neither parents nor experts indicated the need to remove any items; no additional behaviors/skills were identified as missing. Minor wording changes were made to several items following parental feedback.

###### 2.2.1.2.4. Final scale

The final scale consisted of 52 items that were rated on a 5-point Likert scale (0 = Never, 1 = Rarely 2 = Sometimes 3 = Often 4 = Very Often). Parents were instructed to “for each item, please indicate how often over the last week the person shows this behavior, skill, or ability using the response options below.”

#### 2.2.2. Confirmatory sample

In addition to the demographic and diagnostic information questionnaire and the EFS described above, informants completed a comprehensive set of questionnaire measures to evaluate the convergent and discriminant validity of the EFS. These measures included:

Abbreviated version of the Behavior Rating Inventory of Executive Function [BRIEF-sf; (44, 65)]. BRIEF-sf is a 24-item abbreviated version of the standard BRIEF that has demonstrated good reliability and validity across three independent youth samples (65). BRIEF is an informant-report scale designed to capture different aspects of executive functioning. Each item is rated on a 3-point Likert scale (1 = Never, 2 = Sometimes, 3 = Often). The total raw score was used (higher scores mean more severe impairments).

Attention-deficit/hyperactivity disorder assessment [ADHD-ASSESS; (66)]. ADHD-ASSESS is an 18-item informant-report scale designed to capture ADHD symptoms in children aged 2 to 17, including inattention, hyperactivity, and impulsivity. Each item is rated on a 5-point Likert scale A (0 = Never, 1 = Rarely, 2 = Sometimes, 3 = Often, 4 = Very Often). The total raw score was used (higher scores mean more severe impairments).

Comprehensive Anxiety Scale [CAS; (66)]. CAS is a 35-item informant-report scale designed to capture anxiety symptoms in children aged 2 to 17. The instrument provides a total score and six subscale scores covering generalized anxiety, social anxiety, separation anxiety, panic/physiological anxiety symptoms, obsessive/compulsive symptoms, and specific fears. Each item is rated on a 5-point Likert scale (0 = Never, 1 = Rarely, 2 = Sometimes, 3 = Often, 4 = Very Often). This study focused on the total raw score (higher scores mean more severe impairments).

Daily Living Skills Scale [DLSS; (67)]. DLSS is a 53-item informant-report scale designed to capture daily living skills in children aged 2 to 17. The instrument provides a total and three content subscale scores for enhanced interpretation across self-care, home care, and community participation. Each item is rated on a 4-point Likert Scale [0 = Not able to complete (total assistance needed), 1 = Requires significant prompting or assistance, 2 = Requires minimal prompting or assistance, 3 = Completely independent (does not require any assistance or prompting)]. For the current study, we focused on the total raw score (higher scores mean better ability).

### 2.3. Procedure

In Qualtrics, prospective participants reviewed an electronic consent form. Participants who decided to continue with participation indicated consent electronically and began the survey. All participants were paid US$10 for survey completion based on the expected survey completion time (35 min). All data were collected anonymously.

### 2.4. Statistical analyses

Descriptive statistics for demographic and clinical factors were computed to characterize the sample.

#### 2.4.1. Factor structure

Exploratory structural equation models (ESEM) were estimated (68) in the exploratory sample to identify the factor structure of the EFS. These models used weighted least squares mean and variance adjusted estimation, specified four to seven specific factors with an additional general bifactor that included estimation of loadings from all items, and were estimated using geomin rotation. Models were re-estimated in the confirmatory subsample, and the best-fitting model was chosen using a combination of fit statistics and interpretability. Once the best-fitting ESEM model was identified, this model was re-estimated in the total sample, and an equivalent confirmatory ESEM model with all standardized loadings <0.20 set to 0 was estimated. Model fit was evaluated using the comparative fit index (CFI), Tucker–Lewis index (TLI), root mean square error of approximation (RMSEA), and the 95% confidence interval of RMSEA were used to examine model fit (69, 70).

#### 2.4.2. Measurement invariance

The optimal model derived from the factor analyses described above was used as the basis for the evaluation of measurement invariance (71) across age groups (ages 2–4, 5–11, and 12–17 years), sex (male and female), race (Caucasian and other), and ethnicity (Hispanic and non-Hispanic). To examine measurement invariance (equivalence), a series of multi-group confirmatory factor analyses were computed using the theta parameterization and weighted least square mean and variance adjusted (WLSMV) estimation for categorical indicators, following recommended conventions (72) and prior work (73). Model comparisons for measurement invariance analyses were based on empirical work indicating that a drop in CFI or TLI > 0.01 or an increase in RMSEA > 0.01 implies measurement non-equivalence (74, 75).

#### 2.4.3. Reliability

Using the optimal factor model, items with substantive loadings were assigned to scales and classical test theory (CTT) reliability (internal consistency and correct item-total correlations) (76), and item response theory (IRT) analyses were conducted (77) in the entire sample (*n* = 2,004). IRT analyses were conducted separately for each scale as the multi-dimensional bifactor IRT model was not possible to estimate. Analyses used maximum likelihood estimation with robust standard errors, and a logit link with the single factor mean and variance fixed to 0 and 1, respectively. Reliability estimates falling in the ranges 0.70 to 0.79, 0.80 to 0.89, and >0.90 were considered fair, good, and excellent (78). Average corrected item-total correlations ≥0.30 were considered adequate or better (76). Differential item and test functioning were evaluated by examining differences in item characteristic curves and test information curves across age groups, sex, race, and ethnicity.

#### 2.4.4. Convergent and discriminant validity

Convergent and discriminant validity were computed using bivariate correlations (Pearson or Spearman’s non-parametric, where applicable).

## 3. Results

### 3.1. Participant characteristics

The exploratory sample included 2,004 children and adolescents, and the confirmatory sample included 954 children and adolescents. See Table 1 for detailed characteristics across exploratory and confirmatory samples.

### 3.2. Factor structure

**FIGURE 1:**
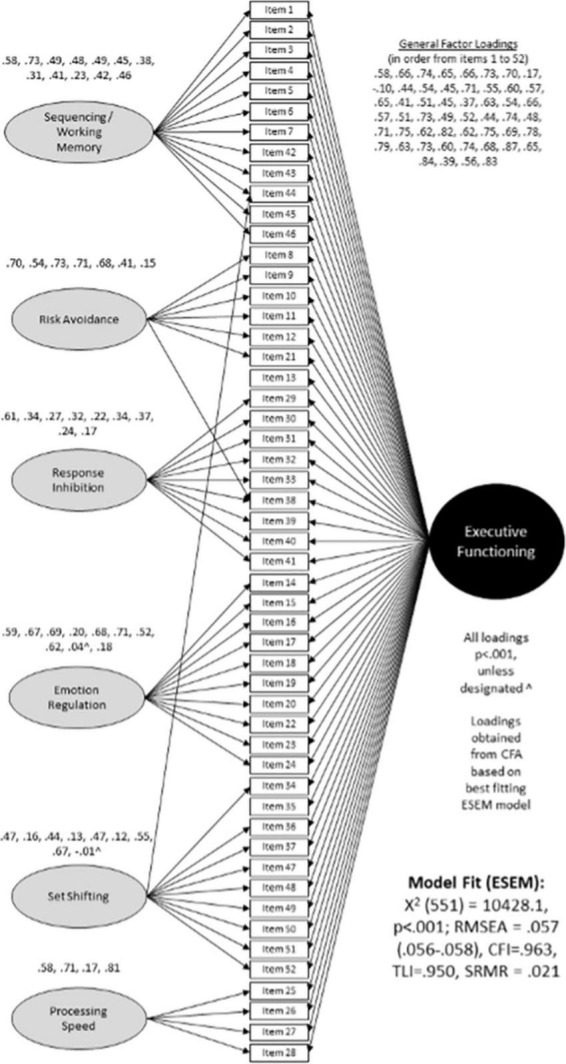
Factor structure of the Executive Functioning Scale (EFS).

In the exploratory and confirmatory samples, ESEM suggested improvements in fit through the six specific factors with a general bifactor ESEM solution. Increases in CFI and TLI and decreases in RMSEA beyond this solution tended to be modest (≤ | 0.006|). The ESEM with seven specific factors and a general executive functioning bifactor had inconsistent difficult to interpret loading patterns across the exploratory and confirmatory subsamples. Thus, the ESEM model with six specific and one general factor was considered the optimal model for additional consideration (Figure 1) (Exploratory sample fit indices: CFI = 0.967, TLI = 0.956, RMSEA = 0.054 [95% CI:0.052,0.055], SRMR = 0.021; Confirmatory sample fit indices: CFI = 0.966, TLI = 0.955, RMSEA = 0.055 [95% CI:0.053,0.056], SRMR = 0.022). A final model was estimated in the total sample (*N* = 2,958) using this model. Fit indices for this model were: CFI = 0.963, TLI = 0.950, RMSEA = 0.057 [95% CI:0.056,0.058], SRMR = 0.021. Given the observation of substantive cross-loadings from items with primary loadings on other factors, a CFA model based on the ESEM model was not estimated. The final scoring was based on the ESEM model, with the subscale choice based on the highest loading for each item. Figure 1 presents the ESEM factor structure of the EFS in the total sample. Identified factors strongly resembled six conceptually based EF constructs of working memory and sequencing (example items: Follows a complete sequence of steps or actions; Can hold several pieces of information in mind at once; Is good at remembering the exact way something happened), response inhibition (example items: Stops what they are doing when told to stop; Focuses on finishing important tasks without being distracted by more interesting activities; Can resist immediate desires because they are not good over the long-term), set-shifting (example items: Can transition from one activity to another without problems; Misses important information because they are engrossed in what they are doing; Has trouble with mentally juggling multiple things) processing speed (example items: Seems to process information slowly; Responds slowly, even when asked to do something they enjoy; Works quickly and accurately on an activity), emotion regulation (example items: Has trouble soothing themselves; Remains upset or emotional longer than others; If they are sad, they seem to have difficulty lifting their mood), and risk avoidance (example items: Does not consider possible danger when doing something; Considers consequences before acting; Seems to crave excitement and new experiences).

### 3.3. Measurement invariance

Estimating measurement invariance for ESEM bifactor models often results in convergence problems. Therefore, a simple confirmatory model without the EF bifactor was used to estimate measurement invariance. This permits examination of measurement equivalence for the subscales with the assumption that if these show scalar invariance, then invariance of the general EF factor is likely. As can be seen from Table 2, results indicated strong (scalar) invariance across age, sex, race, and ethnicity groups.

**TABLE 2:** Measurement invariance analyses for the executive functioning (EF) specific factor model across sex, age, race, and ethnicity.

Sex (M, F)												
Fit							Difference testing					
Model	Par	*X* ^2^	DF	RMSEA	CFI	TLI	*X* ^2^	DF	*p*	ΔRMSEA	ΔCFI	ΔTLI
Configural	607	26721.6	2461	0.082	0.904	0.896	–	–	–	–	–	–
Metric	511	9453.5	2557	0.043	0.973	0.972	85.1	96	0.779	−0.039	0.069	0.076
Scalar	331	11630.5	2737	0.047	0.965	0.966	1269.4	180	<0.0001	0.004	−0.008	−0.006
**Age (2–4, 5–11, and 12–17)**												
Configural	899	25120.7	3703	0.077	0.917	0.911	–	–	–	–	–	–
Metric	707	15692.6	3895	0.055	0.954	0.953	1219.8	192	<0.0001	−0.022	0.037	0.042
Scalar	347	18757.4	4255	0.059	0.944	0.947	4100.1	360	<0.0001	0.004	−0.010	−0.006
**Race (Caucasian, other races)**												
Configural	607	23891.5	2461	0.077	0.915	0.908	–	–	–	–	–	–
Metric	511	9484.4	2557	0.043	0.973	0.971	112.6	96	0.118	−0.034	0.058	0.063
Scalar	331	9453.0	2737	0.041	0.973	0.974	279.3	180	<0.0001	−0.002	0.000	0.003
**Ethnicity (Hispanic, non-Hispanic)**												
Configural	607	21860.2	2461	0.073	0.918	0.911	–	–	–	–	–	–
Metric	511	8447.5	2557	0.039	0.975	0.974	69.5	96	0.981	−0.034	0.057	0.063
Scalar	331	8422.9	2737	0.037	0.976	0.977	217.1	180	0.031	−0.002	0.001	0.003

The observed X^2^ from WLSMV estimated measurement invariance models cannot be directly compared but rather must be compared using difference testing in MPlus. Thus, the apparent reduction from configural to metric models is not an accurate representation of model fit. Instead, the positive X^2^ values from difference testing reflects worse fit of metric relative to configural models.

### 3.4. Reliability

**FIGURE 2:**
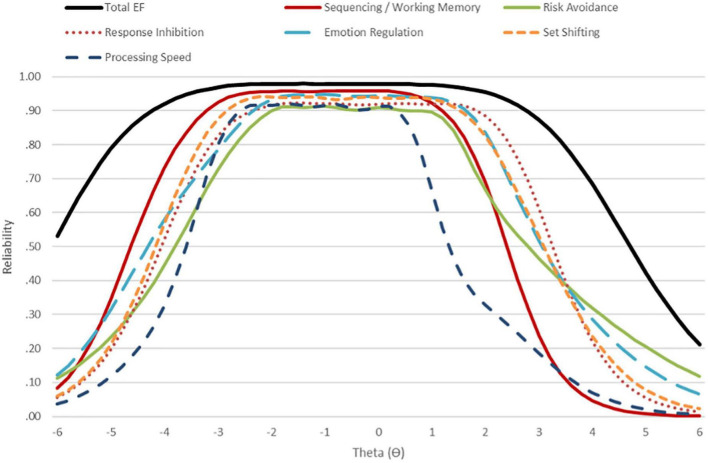
Conditional reliability for the Executive Functioning Scale (EFS) total scale and subscales.

Table 3 shows detailed reliability indices. As can be seen, model reliability was excellent for the general factor (ω = 0.98) and specific factors (ω ≥ 0.89–0.96). Using item scores, internal consistency reliability was excellent for the total scale (α = 0.97) and very good to excellent for all subscale scores (α ≥ 0.84–0.94). Conditional reliability estimates indicated excellent reliability (≥0.90) for the total EF scale from extremely low (θ∼−4.2) to very high (θ∼ + 2.6) scores. Adequate or better reliability (≥0.70) was present for subscale scores in the range from very low (θ∼−3.0) to high scores (∼ + 1.8), except processing speed which showed a drop off in measurement precision beyond high average scores (θ∼ + 0.8) (Figure 2).

**TABLE 3:** Reliability statistics for Executive Functioning Scale (EFS) general (total scores) and specific factors (subscale scores).

	Internal consistency	Model reliability
	α	ω
EF total	0.97	0.98
Sequencing/Working memory	0.94	0.96
Risk avoidance	0.82	0.89
Response inhibition	0.89	0.92
Emotion regulation	0.90	0.93
Set shifting	0.91	0.95
Processing speed	0.84	0.90

Model reliability is McDonald’s omega coefficient derived from bifactor modeling.

### 3.5. Convergent and discriminant validity

The EFS showed strong convergent validity with the 24-item BRIEF-sf (*r* = 0.85) and with the ADHD-ASSESS total score (*r* = −0.76); the latter is relevant because ADHD symptoms include several aspects of cognitive functioning that overlap or are closely related to executive functions, particularly impulsivity (response inhibition). Evidence of discriminant validity with measures of other aspects of functioning and psychopathological symptoms was also good, including associations with DLS (*r* = 0.59) and CAS (*r* = −0.49). Analysis of the pattern of associations with EFS subscales provided further evidence for convergent and discriminant validity. For instance, the EFS ER subscale was significantly more strongly associated with anxiety (measured by CAS; *r* = −0.60) than with daily living skills (measured by DLSS; *r* = 0.35). Table 4 shows the full list of correlations between EFS total and subscale scores with relevant measures used to establish convergent and discriminant validity.

**TABLE 4:** Executive Functioning Scale (EFS) convergent and discriminant validity.

	ADHD-ASSESS	DLS	CAS	BRIEF-sf
EFS total score	−0.76**	0.59**	−0.49**	−0.85**
EFS sequencing/Working memory	−0.61**	0.63**	−0.34**	−0.66**
EFS risk avoidance	−0.54**	0.38**	−0.20**	−0.52**
EFS response inhibition	−0.63**	0.61**	−0.29**	−0.66**
EFS emotion regulation	−0.64**	0.35**	−0.60**	−0.80**
EFS set shifting	−0.74**	0.47**	−0.51**	−0.82**
EFS processing speed	−0.52**	0.28**	−0.42**	−0.79**

*p < 0.01; **p < 0.001; ADHS-ASSESS, attention-deficit/hyperactivity disorder assessment; BRIEF-sf, Behavior Rating Inventory of Executive Functioning, second edition short form; CAS, Comprehensive Anxiety Scale; DLSS, Daily Living Skills Scale; EFS, Executive Functioning Scale.

## 4. Discussion

The EFS is an informant-report measure developed to comprehensively capture a range of crucial executive functioning subdomains relevant across normative and atypical development, including individuals with ASD, other NDD, and NPD. Findings presented in this initial validation demonstrated that the EFS is a psychometrically sound suggesting that it might be a promising instrument for assessing executive functioning across research and clinical contexts. Indeed, the EFS had a clear and replicable factor structure across two independent samples showing strong reliability, good measurement equivalence across age, sex, race, and ethnicity, and preliminary evidence for good convergent and discriminant validity. Crucially, EFS is considerably briefer (52 items) than other dedicated EF measures, such as the BRIEF (86 items).

The EFS was found to have a well-differentiated factor structure that replicated well across exploratory and confirmatory sub-samples. The final model included six specific factors matching the originally hypothesized EF subdomains of working memory and sequencing, response inhibition, set-shifting, processing speed, ER, and risk avoidance, as well as a general EF factor. As noted, there is a wide range of definitions and conceptualizations of EF, some of which emphasize a narrower range of non-affective (“cool”) processes [e.g., (2, 36, 79, 80)] and others arguing for a broader conceptualization that also includes affective (“hot”) constructs (37, 39, 41). Although most disorders are associated with relatively uniform impairments across specific constructs of “cool” EF, there are pronounced variations in effect sizes of specific EF deficits in certain conditions (14). Similarly, different NDD and NPD have been suggested to show distinct profiles of “hot” EF subdomains, including ER and risk avoidance (41). Thus, it is essential to fully capture different EF subdomains to understand whether specific subdomains might be more strongly associated with particular aspects of psychopathology.

Several existing instruments, including the BRIEF, CEFI, and BDEFS, have been designed explicitly for assessing deficits in different aspects of EF in NDD and NPD. However, as noted, certain domain coverage and representation limitations, such as lack of coverage of risk avoidance/taking and upregulation of positive emotions, restrict their utility for comprehensive characterization of EF. Further, a key assumption that must be met for widespread measure adoption across diverse sex, age, race, ethnicity, and clinical groups is demonstrated invariance; however, there is little evidence for the invariance of the existing scales. Moreover, the majority of existing EF instruments lacks evidence for conditional reliability, a key feature necessary for capturing and tracking very high and very low levels of a particular trait with good precision. Conversely, the best-fitting EFS model was consistent across sex, age, race, and ethnicity groups, indicating that it can be interpreted consistently when implemented across diverse demographics. Further, conditional reliability estimates showed excellent reliability (≥0.90) for the total EFS from extremely low to very high scores and at least adequate reliability (≥0.70) for subscales from very low to high scores.

Despite the strengths of this two-sample development and validation approach, several limitations are important to note. The main limitation of this study was a reliance on informant reports, including diagnoses, cognitive levels, and symptom severity estimates. Given the online nature of the research and the need to collect large sample sizes across both exploratory and confirmatory samples, it was not possible to independently confirm the diagnostic status of participants and administer gold-standard diagnostic assessments, including the Autism Diagnostic Observation Schedule and the Autism Diagnostic Interview-Revised and dedicated cognitive assessments. However, it is essential to note that high rates of verification of ASD from clinical reports (81, 82) and high concordance (>97%) with clinician best estimate diagnoses and with standardized instruments (83) have been shown across prior online studies collecting parent-reported diagnoses. It has also been demonstrated that parent-report of children’s IQ strongly correlates with standardized clinical IQ testing [e.g., (84)]. In addition, given that the current study relied on parent-reported clinical information only to describe the sample, we believe that these variables are an adequate proxy at the psychometric evaluation stage. However, it will be important for future studies to further evaluate the factor structure and psychometric properties of the EFS in large samples of individuals with discrete categorically defined neurodevelopmental and neuropsychiatric diagnoses established based on the gold-standard diagnostic instruments and clinical consensus. The present study was further limited by the lack of a more comprehensive set of questionnaires and performance-based EF assessments and by the relatively small sample of individuals with ASD. Thus, given the described limitations, it will be crucial for future studies to further validate the EFS in clinical settings conducive to detailed in-person observational and performance-based assessments and, ideally, utilize longitudinal designs to explore the predictive validity of the EFS. Finally, given dynamic and non-linear changes in the manifestation and complexity of specific facets of EF across different stages of development, it will be important for future studies to provide more detailed testing of the EFS performance across different periods of development, in particular during first 5 years of life, and, where relevant, develop further items to capture developmentally sensitive and specific instances and manifestations of the EF. Although not a limitation *per se*, it is important to highlight the fact that EFS captures several constructs, including emotion regulation and processing speed, that are not included in all of the existing EF models. However, given that there is no universally accepted EF taxonomy and given the high clinical relevance of noted constructs across a range of neurodevelopmental and neuropsychiatric conditions, EFS was designed to provide a comprehensive capture of a broader range of EF-related constructs.

In summary, despite the noted limitations, the present data provide preliminary evidence that the EFS is a free, relatively brief, open-source, valid, and reliable measure for the comprehensive characterization of distinct subdomains of executive functioning that are relevant for the understanding of individual differences in clinical outcomes across a range of NDD and NPD. Further, EFS shows excellent measurement precision for capturing a wide range of abilities, which suggests a tremendous potential for its use for treatment tracking. Thus, with further replication, the EFS has excellent potential for wide adoption across research and clinical contexts.

## Data availability statement

The raw data supporting the conclusions of this article will be made available by the authors, without undue reservation.

## Ethics statement

This studies involving human participants were reviewed and approved by John Carroll University Institutional Review Board. Written informed consent to participate in this study was provided by the participants’ legal guardian/next of kin.

## Author contributions

TF, MU, and AH designed the study. TF and MU collected the data, had full access to the data, and conducted the analyses. MU, TF, RC, and AH drafted the initial manuscript. All authors critically reviewed and provided the feedback on the initial version of manuscript and approved the final version of the manuscript.

## Footnotes

1
https://prolific.co/

## References

[1] Barkley RA. The assessment of executive functioning using the Barkley deficits in executive functioning scales. In: Goldstein S Naglieri JA editors. *Handbook of Executive Functioning.* Berlin: Springer Science + Business Media (2014). p. 245–63. 10.1007/978-1-4614-8106-5_15

[2] Diamond A. Executive functions. *Annu Rev Psychol.* (2013) 64:135–68. 10.1146/annurev-psych-113011-143750 23020641 PMC4084861

[3] Gross JJ. Emotion regulation: current status and future prospects. *Psychol Inquiry.* (2015) 26:1–26. 10.1080/1047840X.2014.940781

[4] Kochanska G Murray K Coy KC. Inhibitory control as a contributor to conscience in childhood: from toddler to early school age. *Child Dev.* (1997) 68:263–77. 10.1111/j.1467-8624.1997.tb01939.x 9180001

[5] Rothbart MK Ellis LK Posner MI. Temperament and self-regulation. In: Vohs KD Baumeister RF editors. *Handbook of Self-Regulation: Research, Theory, and Applications.* New York, NY: The Guilford Press (2011). p. 441–60.

[6] Tangney JP Baumeister RF Boone AL. High self-control predicts good adjustment, less pathology, better grades, and interpersonal success. *J Pers.* (2004) 72:271–324. 10.1111/j.0022-3506.2004.00263.x 15016066

[7] Duncan GJ Dowsett CJ Claessens A Magnuson K Huston AC Klebanov P School readiness and later achievement. *Dev Psychol.* (2007) 43:1428–46. 10.1037/0012-1649.43.6.1428 18020822

[8] Baker S Morawska A Mitchell A. Promoting children’s healthy habits through self-regulation via parenting. *Clin Child Family Psychol Rev.* (2019) 22:52–62. 10.1007/s10567-019-00280-6 30725307

[9] Davis JC Marra CA Najafzadeh M Liu-Ambrose T. The independent contribution of executive functions to health related quality of life in older women. *BMC Geriatr.* (2010) 10:16. 10.1186/1471-2318-10-16 20359355 PMC2867806

[10] Cai RY Hardan AY Phillips JM Frazier TW Uljarević M. Brief report: emotion regulation influences on internalizing and externalizing symptoms across the normative-clinical continuum. *Front Psychiatry.* (2021) 12:693570. 10.3389/fpsyt.2021.693570 34366922 PMC8333703

[11] Etkin A Gyurak A O’Hara R. A neurobiological approach to the cognitive deficits of psychiatric disorders. *Dialog Clin Neurosci.* (2013) 15:419–29. 10.31887/DCNS.2013.15.4/aetkin PMC3898680 24459409

[12] Goschke T. Dysfunctions of decision-making and cognitive control as transdiagnostic mechanisms of mental disorders: advances, gaps, and needs in current research. *Int J Methods Psychiatr Res.* (2014) 23(Suppl. 1):41–57. 10.1002/mpr.1410 24375535 PMC6878557

[13] McTeague LM Goodkind MS Etkin A. Transdiagnostic impairment in cognitive control neurocircuits: behaviour, structure, and function. In: Egner T editor. *The Wiley Handbook of Cognitive Control.* New York, NY: Wiley Blackwell (2017). p. 539–55. 10.1002/9781118920497.ch30

[14] Snyder HR Miyake A Hankin BL. Advancing understanding of executive function impairments and psychopathology: bridging the gap between clinical and cognitive approaches. *Front. Psychol.* (2015) 6:328. 10.3389/fpsyg.2015.00328 25859234 PMC4374537

[15] Cai RY Richdale AL Uljarević M Dissanayake C Samson AC. Emotion regulation in autism spectrum disorder: where we are and where we need to go. *Autism Res.* (2018) 11:962–78. 10.1002/aur.1968 29979494

[16] Wallace GL Yerys BE Peng C Dlugi C Anthony LG Kenworthy L. Chapter three – assessment and treatment of executive function impairments in autism spectrum disorder: an update. *Int Rev Res Dev Disabil.* (2016) 51:85–122. 10.1016/bs.irrdd.2016.07.004

[17] Alderson RM Kasper LJ Hudec KL Patros CH. Attention-deficit/hyperactivity disorder (ADHD) and working memory in adults: a meta-analytic review. *Neuropsychology.* (2013) 27:287–302. 10.1037/a0032371 23688211

[18] Faraone SV Rostain AL Blader J Busch B Childress AC Connor DF Practitioner review: emotional dysregulation in attention-deficit/hyperactivity disorder – implications for clinical recognition and intervention. *J Child Psychol Psychiatry Allied Discip.* (2019) 60:133–50. 10.1111/jcpp.12899 29624671

[19] Forbes NF Carrick LA McIntosh AM Lawrie SM. Working memory in schizophrenia: a meta-analysis. *Psychol Med.* (2009) 39:889–905. 10.1017/S0033291708004558 18945379

[20] Stefanopoulou E Manoharan A Landau S Geddes JR Goodwin G Frangou S. Cognitive functioning in patients with affective disorders and schizophrenia: a meta-analysis. *Int Rev Psychiatry.* (2009) 21:336–56. 10.1080/09540260902962149 20374148

[21] Rock PL Roiser JP Riedel WJ Blackwell AD. Cognitive impairment in depression: a systematic review and meta-analysis. *Psychol Med.* (2014) 44:2029–40. 10.1017/S0033291713002535 24168753

[22] Snyder HR. Major depressive disorder is associated with broad impairments on neuropsychological measures of executive function: a meta-analysis and review. *Psychol Bull.* (2013) 139:81–132. 10.1037/a0028727 22642228 PMC3436964

[23] Abramovitch A Abramowitz JS Mittelman A. The neuropsychology of adult obsessive-compulsive disorder: a meta-analysis. *Clin Psychol Rev.* (2013) 33:1163–71. 10.1016/j.cpr.2013.09.004 24128603

[24] Shin NY Lee TY Kim E Kwon JS. Cognitive functioning in obsessive-compulsive disorder: a meta-analysis. *Psychol Med.* (2014) 44:1121–30. 10.1017/S0033291713001803 23866289

[25] Polak AR Witteveen AB Reitsma JB Olff M. The role of executive function in posttraumatic stress disorder: a systematic review. *J Affect Disord.* (2012) 141:11–21. 10.1016/j.jad.2012.01.001 22310036

[26] Hollocks MJ Jones CR Pickles A Baird G Happé F Charman T The association between social cognition and executive functioning and symptoms of anxiety and depression in adolescents with autism spectrum disorders. *Autism Res.* (2014) 7:216–28. 10.1002/aur.1361 24737743

[27] Uljarević M Richdale AL Evans DW Cai RY Leekam SR. Interrelationship between insistence on sameness, effortful control and anxiety in adolescents and young adults with autism spectrum disorder (ASD). *Mol Autism.* (2017) 8:36. 10.1186/s13229-017-0158-4 28736608 PMC5521115

[28] Uljarević M Frazier TW Rached G Busch RM Klaas P Srivastava S Brief report: role of parent-reported executive functioning and anxiety in insistence on sameness in individuals with Germline PTEN mutations. *J Autism Dev Disord.* (2022) 52:414–22. 10.1007/s10803-021-04881-5 33595755 PMC8479547

[29] Leung RC Vogan VM Powell TL Anagnostou E Taylor MJ. The role of executive functions in social impairment in autism spectrum disorder. *Child Neuropsychol.* (2016) 22:336–44. 10.1080/09297049.2015.1005066 25731979

[30] Lieb RW Bohnert AM. Relations between executive functions, social impairment, and friendship quality on adjustment among high functioning youth with autism spectrum disorder. *J Autism Dev Disord.* (2017) 47:2861–72. 10.1007/s10803-017-3205-2 28624964

[31] Horne CM Sahni A Pang SW Vanes LD Szentgyorgyi T Averbeck B The role of cognitive control in the positive symptoms of psychosis. *Neuroimage Clin.* (2022) 34:103004. 10.1016/j.nicl.2022.103004 35468567 PMC9059151

[32] Orellana G Slachevsky A. Executive functioning in schizophrenia. *Front Psychiatry.* (2013) 4:35. 10.3389/fpsyt.2013.00035 23805107 PMC3690455

[33] Ruiz-Castañeda P Santiago-Molina E Aguirre-Loaiza H Daza González MT. “Cool” and “Hot” executive functions in patients with a predominance of negative schizophrenic symptoms. *Front Psychol.* (2020) 11:571271. 10.3389/fpsyg.2020.571271 33250814 PMC7674804

[34] Maddox BB Cleary P Kuschner ES Miller JS Armour AC Guy L Lagging skills contribute to challenging behaviors in children with autism spectrum disorder without intellectual disability. *Autism.* (2018) 22:898–906. 10.1177/1362361317712651 28844152 PMC6113117

[35] Miyake A Friedman NP. The nature and organization of individual differences in executive functions: four general conclusions. *Curr Dir Psychol Sci.* (2012) 21:8–14. 10.1177/0963721411429458 22773897 PMC3388901

[36] Goldstein S Naglieri JA Princiotta D Otero TM. *Introduction: A History of Executive Functioning as a Theoretical and Clinical Construct. In: Handbook of Executive Functioning.* New York, NY: Springer (2014). p. 3–12. 10.1007/978-1-4614-8106-5_1

[37] Zelazo PD Carlson SM. Hot and cool executive function in childhood and adolescence: development and plasticity. *Child Dev Perspect.* (2012) 6:354–60. 10.1111/j.1750-8606.2012.00246.x

[38] Gross JJ Jazaieri H. Emotion, emotion regulation, and psychopathology: an affective science perspective. *Clin Psychol Sci.* (2014) 2:387–401. 10.1177/2167702614536164

[39] Casey BJ. Beyond simple models of self-control to circuit-based accounts of adolescent behavior. *Annu Rev Psychol.* (2015) 66:295–319. 10.1146/annurev-psych-010814-015156 25089362

[40] Ernst M. The triadic model perspective for the study of adolescent motivated behavior. *Brain Cogn.* (2014) 89:104–11. 10.1016/j.bandc.2014.01.006 24556507 PMC4248307

[41] Nigg JT. Annual research review: on the relations among self-regulation, self-control, executive functioning, effortful control, cognitive control, impulsivity, risk-taking, and inhibition for developmental psychopathology. *J Child Psychol Psychiatry Allied Discip.* (2017) 58:361–83. 10.1111/jcpp.12675 28035675 PMC5367959

[42] McGrath LM Braaten EB Doty ND Willoughby BL Wilson HK O’Donnell EH Extending the ‘cross-disorder’ relevance of executive functions to dimensional neuropsychiatric traits in youth. *J Child Psychol Psychiatry.* (2015) 57:462–71. 10.1111/jcpp.12463 26411927 PMC4876048

[43] Nigg JT Jester JM Stavro GM Ip KI Puttler LI Zucker RA. Specificity of executive functioning and processing speed problems in common psychopathology. *Neuropsychology.* (2017) 31:448–66. 10.1037/neu0000343 28094999 PMC5408314

[44] Gioia GA Isquith PK Guy SC Kenworthy L. *Behavior Rating Inventory of Executive Function^®^, Second Edition (BRIEF^®^ 2).* Lutz, FL: PAR Inc (2015).

[45] Naglieri JA Goldstein S. Using the comprehensive executive function inventory (CEFI) to assess executive function: from theory to application. In: Goldstein S Naglieri J editors. *Handbook of Executive Functioning.* New York, NY: Springer (2014). 10.1007/978-1-4614-8106-5_14

[46] Barkley RA. *Barkley Deficits in Executive Functioning Scale (BDEFS for Adults).* New York, NY: Guilford Press (2011).

[47] Barkley RA Murphy KR. The nature of executive function (EF) deficits in daily life activities in adults with ADHD and their relationship to performance on EF tests. *J Psychopathol Behav Assess.* (2011) 33:137–58. 10.1007/s10862-011-9217-x

[48] Barkley RA. *Barkley Deficits in Executive Functioning Scale–Children and Adolescents (BDEFS-CA).* New York, NY: Guilford Press (2012).

[49] Delis DC Kaplan E Kramer JH. *Delis-Kaplan Executive Function System (D–KEFS) [Database Record].* San Antonio, TX: APA PsycTests. (2001). 10.1037/t15082-000

[50] Cambridge Cognition. *Neuropsychological Test Automated Battery (CANTABeclipse) Manual.* Cambridge: Cambridge Cognition Limited (2006).

[51] Korkman M Kirk U Kemp S. *NEPSY-II.* 2nd ed. San Antonio, TX: Harcourt Assessment (2007).

[52] Zelazo PD Anderson JE Richler J Wallner-Allen K Beaumont JL Conway KP NIH toolbox cognition battery (CB): validation of executive function measures in adults. *J Int Neuropsychol Soc.* (2014) 20:620–9. 10.1017/S1355617714000472 24960301 PMC4601803

[53] Fernández-Alcántara M Albaladejo-Blázquez N Fernández-Ávalos MI Sánchez-SanSegundo M Cruz-Quintana F Pérez-Martínez V Validity of the computerized battery for neuropsychological evaluation of children (BENCI) in Spanish children: preliminary results. *Eur J Investig Health Psychol Educ.* (2022) 12:893–903. 10.3390/ejihpe12080065 35893081 PMC9330040

[54] Eisenberg N Spinrad TL Eggum ND. Emotion-related self-regulation and its relation to children’s maladjustment. *Annu Rev Clin Psychol.* (2010) 6:495–525. 10.1146/annurev.clinpsy.121208.131208 20192797 PMC3018741

[55] Carl JR Soskin DP Kerns C Barlow DH. Positive emotion regulation in emotional disorders: a theoretical review. *Clin Psychol Rev.* (2013) 33:343–60. 23399829 10.1016/j.cpr.2013.01.003

[56] Gross JJ John OP. Individual differences in two emotion regulation processes: implications for affect, relationships, and well-being. *J Pers Soc Psychol.* (2003) 85:348–62. 10.1037/0022-3514.85.2.348 12916575

[57] Ng TH Stange JP Black CL Titone MK Weiss RB Abramson LY Impulsivity predicts the onset of DSM-IV-TR or RDC hypomanic and manic episodes in adolescents and young adults with high or moderate reward sensitivity. *J Affect Disord.* (2016) 198:88–95. 10.1016/j.jad.2016.03.045 27011364 PMC4844858

[58] Jimenez A Lucas-Molina B. Dimensional structure and measurement invariance of the BRIEF-2 across gender in a socially vulnerable sample of primary school-aged children. *Child Neuropsychol.* (2019) 25:636–47. 10.1080/09297049.2018.1512962 30175686

[59] Marjanovic Z Bajkov L MacDonald J. The conscientious responders scale helps researchers verify the integrity of personality questionnaire data. *Psychol Rep.* (2019) 122:1529–49. 10.1177/0033294118783917 29914343

[60] Reynolds CR Kamphaus RW. *Behavior Assessment System for Children.* 3rd ed. London: Pearson (2015). 10.1002/9781118625392.wbecp447

[61] Briggs-Gowan MJ Carter AS Irwin JR Wachtel K Cicchetti DV. The brief infant-toddler social and emotional assessment: screening for social-emotional problems and delays in competence. *J Pediatr Psychol.* (2004) 29:143–55. 10.1093/jpepsy/jsh017 15096535

[62] Gartstein MA Rothbart MK. Studying infant temperament via the revised infant behavior questionnaire. *Infant Behav Dev.* (2003) 26:64–86. 10.1016/S0163-6383(02)00169-8

[63] Shields A Cicchetti D. *Emotion Regulation Checklist.* Indianapolis, IN: APA PsycTests (1995). 10.1037/t08132-000

[64] Gratz KL Roemer L. Multidimensional assessment of emotion regulation and dysregulation: development, factor structure, and initial validation of the difficulties in emotion regulation scale. *J Psychopathol Behav Assess.* (2004) 26:41–54. 10.1023/B:JOBA.0000007455.08539.94

[65] LeJeune B Beebe D Noll J Kenealy L Isquith P Gioia G. Psychometric support for an abbreviated version of the behavior rating inventory of executive function (BRIEF) parent form. *Child Neuropsychol.* (2010) 16:182–201. 10.1080/09297040903352556 20029694

[66] Spackman E Uljarević M Hardan AY Frazier TW. Characterizing attention-deficit/hyperactivity disorder, anxiety and mood symptoms in youth with autism and other neurodevelopmental and neuropsychiatric conditions: development and validation of new assessment battery. In: *Poster Presented at the San Francisco Bay Area Conference.* San Francisco, CA (2022).

[67] Frazier TW Spackman E Hardan AY Uljarević M. Development and validation of a new open source measure of daily living skills. In: *Poster Presented at the San Francisco Bay Area Conference.* San Francisco, CA (2022).

[68] Marsh HW Morin AJ Parker PD Kaur G. Exploratory structural equation modeling: an integration of the best features of exploratory and confirmatory factor analysis. *Annu Rev Clin Psychol.* (2014) 10:85–110. 10.1146/annurev-clinpsy-032813-153700 24313568

[69] Hu L Bentler PM. Cutoff criteria for fit indexes in covariance structure analysis: conventional criteria versus new alternatives. *Struct Equ Model.* (1999) 6:1–55. 10.1080/10705519909540118

[70] Marsh HW Hau K-T Wen Z. In search of golden rules: comment on hypothesis-testing approaches to setting cutoff values for fit indexes and dangers in overgeneralizing Hu and Bentler’s (1999) findings. *Struct Equ Model.* (2004) 11:320–41. 10.1207/s15328007sem1103_2

[71] Chiorri C Marsh HW Ubbiali A Donati D. Testing the factor structure and measurement invariance across gender of the big five inventory through exploratory structural equation modeling. *J Pers Assess.* (2016) 98:88–99. 10.1080/00223891.2015.1035381 25932664

[72] Putnick DL Bornstein MH. Measurement invariance conventions and reporting: the state of the art and future directions for psychological research. *Dev Rev.* (2016) 41:71–90. 10.1016/j.dr.2016.06.004 27942093 PMC5145197

[73] Frazier TW Hardan AY. Equivalence of symptom dimensions in females and males with autism. *Autism.* (2017) 21:749–59. 10.1177/1362361316660066 27503465

[74] Chen FF. Sensitivity of goodness of fit indexes to lack of measurement invariance. *Struct Equ Model.* (2007) 14:464–504. 10.1080/10705510701301834

[75] Cheung GW Rensvold RB. Evaluating goodness-of-fit indexes for testing measurement invariance. *Struct Equ Model.* (2002) 9:233–55. 10.1207/S15328007SEM0902_5 33486653

[76] Streiner D Norman G. *Health Measurement Scales: a Practical Guide to Their Development and Use.* 2nd ed. Oxford: Oxford University Press (1995).

[77] Embretson SE Reise SP. *Item Response Theory for Psychologists.* Mahwah, NJ: Lawrence Erlbaum Associates Publishers (2000).

[78] Nunnally JC Bernstein IH. The assessment of reliability. *Psychometr Theor.* (1994) 3:248–92.

[79] Collins A Koechlin E Reasoning, learning, and creativity: frontal lobe function and human decision-making. *PLoS Biol.* (2012) 10:e1001293. 10.1371/journal.pbio.1001293 22479152 PMC3313946

[80] Lunt L Bramham J Morris RG Bullock PR Selway RP Xenitidis K Prefrontal cortex dysfunction and ‘Jumping to Conclusions’: bias or deficit? *J Neuropsychol.* (2012) 6:65–78. 10.1111/j.1748-6653.2011.02005.x 22257612

[81] Daniels AM Rosenberg RE Anderson C Law JK Marvin AR Law PA. Verification of parent-report of child autism spectrum disorder diagnosis to a web-based autism registry. *J Autism Dev Disord.* (2012) 42:257–65. 10.1007/s10803-011-1236-7 21468770

[82] Feliciano P Zhou X Astrovskaya I Turner TN Wang T Brueggeman L Exome sequencing of 457 autism families recruited online provides evidence for autism risk genes. *NPJ Genom Med.* (2019) 4:19. 10.1038/s41525-019-0093-8 31452935 PMC6707204

[83] Lee H Marvin AR Watson T Piggot J Law JK Law PA Accuracy of phenotyping of autistic children based on Internet implemented parent report. *Am J Med Genet B Neuropsychiatr Genet.* (2010) 153B:1119–26. 10.1002/ajmg.b.31103 20552678 PMC4311721

[84] Shu C Green Snyder L Shen Y Chung WK SPARK Consortium. Imputing cognitive impairment in SPARK, a large autism cohort. *Autism Res.* (2022) 15:156–70. 10.1002/aur.2622 34636158

